# A commentary on the article ‘Family caregiver outcomes after participating in a hospital-based family involvement program after major gastrointestinal surgery: a subgroup analysis of a patient preferred cohort study’

**DOI:** 10.1097/JS9.0000000000001596

**Published:** 2024-05-10

**Authors:** Yajie Wang, Tan Tan, Yisheng Pan

**Affiliations:** Department of Gastrointestinal Surgery, Peking University First Hospital, Beijing, People’s Republic of China

*Dear Editor,*

We were interested to read the impressive study by Musters *et al*.^1^ in the *International Journal of Surgery* and would like to congratulate the authors for their interesting findings. In this great work, the authors evaluated family caregiver outcomes after participation in a hospital-based family intervention program (FIP) following a patient’s major gastrointestinal surgery. They assessed family caregivers’ burden and their well-being up to 90 days after discharge. The family caregivers of oncological surgical patients who participated in the FIP exhibited acceptable caregiver burden and well-being levels. However, we wish to discuss the following concerns.

With the increasing incidence of colorectal cancer, more and more patients are undergoing enterostomy, including temporary ileostomy and permanent enterostomy, often for fecal metastasis after low anterior resection. According to statistics, the number of patients requiring permanent enterostomy is increasing year by year^2^. Often, the body image, bowel patterns, and lifestyle habits of patients undergoing an enterostomy to survive are dramatically changed, which has a seriously different impact on the patient’s physical and mental health^3,4^. Similarly, problems with involuntary defecation, exposure of the intestinal mucosa, stool leakage, and unpleasant odors that occur in permanent enterostomy patients are also common for caregivers of such patients to face more stress and serious interference with their quality of life^5,6^. In addition, some patients (such as amyotrophic lateral sclerosis patients) also need to be operated with gastrostomy surgery to ensure food, which is not a small challenge for family care. A study from China demonstrated caregivers’ health was affected by their self-perceived health status and occupation, the presence of other family members with caregiving needs, patients’ expected stoma appliance wear time, and stoma self-care status^7^. Moreover, the longer stoma appliances’ wear time increases the caregiver’s perceived stress, which means that ostomy care is bound to affect the well-being of family caregivers. In this paper, the author mentions home care after gastrointestinal surgery, and we believe that it is necessary to discuss the existence of stoma as a very important topic. To sum up, it is necessary to consider the presence or absence of ostomy care as an adjustment factor of the caregiver’s burden and well-being levels to ensure more accurate results.

Finally, we truly thank the authors for their excellent and important work.

## Ethical approval

Because this is a commentary of a research paper, ethical approval is not required.

## Consent

Because this is a commentary of a research paper, consent is not required.

## Source of funding

None.

## Author contribution

Y.W.: literature collection and manuscript writing; T.T.: literature collection and manuscript revised; Y.P.: project development and manuscript revised.

## Conflicts of interest disclosure

All authors declare that they do not have any conflicts of interest.

## Research registration unique identifying number (UIN)

Because this is a commentary of a research paper, research registration is not required.

## Guarantor

Yisheng Pan.

## Data availability statement

This manuscript is a comment. All the data from the current study are publicly available.

## Provenance and peer review

Our paper was not invited.
